# Wine or Beer? Comparison, Changes and Improvement of Polyphenolic Compounds during Technological Phases

**DOI:** 10.3390/molecules25214960

**Published:** 2020-10-27

**Authors:** Sanja Radonjić, Vesna Maraš, Jovana Raičević, Tatjana Košmerl

**Affiliations:** 1 “13. Jul Plantaže” a.d., Research and Development Sector, Put Radomira Ivanovića 2, 81000 Podgorica, Montenegro; vesnam@t-com.me (V.M.); jovana_raicevic@yahoo.com (J.R.); 2 Biotechnical Faculty, University of Ljubljana, Jamnikarjeva 101, 1000 Ljubljana, Slovenia; tatjana.kosmerl@bf.uni-lj.si

**Keywords:** wine, beer, polyphenols, antioxidant activity, winemaking, brewing

## Abstract

Wine and beer are nowadays the most popular alcoholic beverages, and the benefits of their moderate consumption have been extensively supported by the scientific community. The main source of wine and beer’s antioxidant behavior are the phenolic substances. Phenolic compounds in wine and beer also influence final product quality, in terms of color, flavor, fragrance, stability, and clarity. Change in the quantity and quality of phenolic compounds in wine and beer depends on many parameters, beginning with the used raw material, its place of origin, environmental growing conditions, and on all the applied technological processes and the storage of the final product. This review represents current knowledge of phenolic compounds, comparing qualitative and quantitative profiles in wine and beer, changes of these compounds through all phases of wine and beer production are discussed, as well as the possibilities for increasing their content. Analytical methods and their importance for phenolic compound determination have also been pointed out. The observed data showed wine as the beverage with a more potent biological activity, due to a higher content of phenolic compounds. However, both of them contain, partly similar and different, phenolic compounds, and recommendations have to consider the drinking pattern, consumed quantity, and individual preferences. Furthermore, novel technologies have been developing rapidly in order to improve the polyphenolic content and antioxidant activity of these two beverages, particularly in the brewing industry.

## 1. Introduction

Wine has existed on Earth for more than 6000 years [1], while beer has existed for over 5000 years [2]. Throughout history, both drinks were produced in Ancient Egypt and regions of Mesopotamia. Wine was used in various therapies and treatments, while beer was an essential part of diet, first to appear when people began agriculture. The brewing industry is more linked to northern Europe, where due to cold conditions viticulture development was inhibited. Both of these beverages are very complex in terms of their ingredients, and besides their long traditions, there are so many characteristics and parameters that determine their final quality, from the quality of raw material (malt and hop for beer and grape for wine), yeast, regimes of alcoholic fermentation, conditions of aging etc. However, the parameters of all the phases of production and composition of these two beverages have been very well studied by many researchers, since the early 20th century. Besides their flavor, which determines their use, wine and beer are known as rich with bioactive compounds, i.e., antioxidants that increase the interest in their nutritional profile. A great number of studies and comprehensive reviews have dealt with the bioactive compounds responsible for the possible health benefits due to moderate wine and beer consumption, and with the different methods of improvement of the antioxidant compounds in these two beverages [3,4,5,6,7,8,9,10,11,12,13,14]. Much of this research supports the thesis that moderate consumption of alcoholic beverages, such are red wine and beer, positively influences the decrease of cardiovascular disease [3]. Key roles as antioxidants in wine and beer belong to the phenolic compounds, and many of them, such as flavonoids, have an effect on cardiovascular and chronic degenerative diseases [15,16], non-flavonoids (stilbenes, hydroxycinnamic, and hydroxybenzoic acids) also positively affect the cardiovascular system [17]. In addition, it has been recently shown that there is a relation between beer consumption and higher protection against coronary diseases, compared to other spirits, and beer is also associated with bone density increase, and with immunological and cardiovascular benefits [18,19,20]. However, there are huge differences between the phenolic profile and content among red wine and beer, primarily due to the different raw material used in their production. The importance of phenolic compounds for wine and beer is very significant, as their presence influences the final quality of these products. Some polyphenol classes can only be found in beer (chalcones and flavanones) and others are mainly found in wine (stilbenes, proanthocyanidins), while flavanols and flavan-3-ols are found in similar concentrations in both beverages. In beer quality they play a key role, as they influence the time of transport and storage, flavor stability, clarity, and color of beer. Additionally, phenolic compounds are essential in wine, because they determine the sensorial wine characteristics (taste and fragrance), color, microbiological and oxidative stability, and chemical properties of wine, as they interact with other compounds including other polyphenols, proteins, and polysaccharides.

Production of wine and beer consists of many technological phases, which are influenced by many parameters, and the huge numbers of occurring variables; the changes in biochemistry are very complex. In both beverages, the composition of phenolic compounds is very diverse and depends on many similar parameters, first of all on the genetic factors of the raw material and the environmental conditions during their growth, as well as technological and aging factors [21]. In regards to beer, malt and hops represent the two main ingredients on which antioxidant compounds depend; actually 70–80% are derived from malt, and the remaining from hops [22,23], and this ratio also depends on the type of the beer [24]. Furthermore, during beer making, important technological phases, in which the change of polyphenolic compounds occurs, begins with the malting process (steeping and germination), kilning, mashing, wort separation and boiling, whirlpool rest, through to the fermentation, maturation, and at the end, to the stabilization/filtration and bottling. Primarily classification of beer is made based on the fermentation process [25], and in these terms there are lagers, ales, and lambic types of beer. The most consumed are lagers, produced by low fermentation at lower temperatures (6–15 °C), while in contrast ale-type beer is made by high fermentation at higher temperatures between 16–24 °C, and as a result of spontaneous fermentation there is lambic beer. Exclusively, grape is used as the raw material for wine production, and based on the color of the used grape varieties, wine is classified into red and white. The main difference, and at the same time the most important, between the making of red and white is that during the making of red, along with alcoholic fermentation, maceration i.e., extraction of color and other substances from grape skin and seed occurs, while within the process of the alcoholic fermentation of whites only colorless and clarified grape juice is used in the process of alcoholic fermentation. As for making rose wine, winemakers use limited skin contact in order to extract color and some compounds, depending on the desired degree of complexity. Due to this maceration, occurring along with alcoholic fermentation during red winemaking, in which the phenolic compounds are extracted from grapes, this step represents the key one in determining the content of polyphenolic compounds in red wine. Furthermore, because of this step, it is commonly known that red wine contains more antioxidant compounds, and has been more studied and reviewed by researchers in the last decades [14,26,27,28,29,30,31,32,33,34]. Another important step during winemaking in which it is possible to increase polyphenolic compounds is ageing in wood barrels, or with addition of oak alternatives.

Considering the fact that the beer and wine markets are becoming more competitive and saturated, and considering that consumers nowadays are more interested in beverages influencing in positive way their health, novel technologies for beer and wine are developing in order to produce products with higher antioxidant potential, as well with special personality. The aim of this review is to present the current state of the knowledge of phenolic compounds in beer and wine as well as the possibilities of their increase during different technological phases in beer and wine production, in moving from the raw material to the final product.

## 2. Bioactive Compounds in Beer and Wine

**Figure 1:**
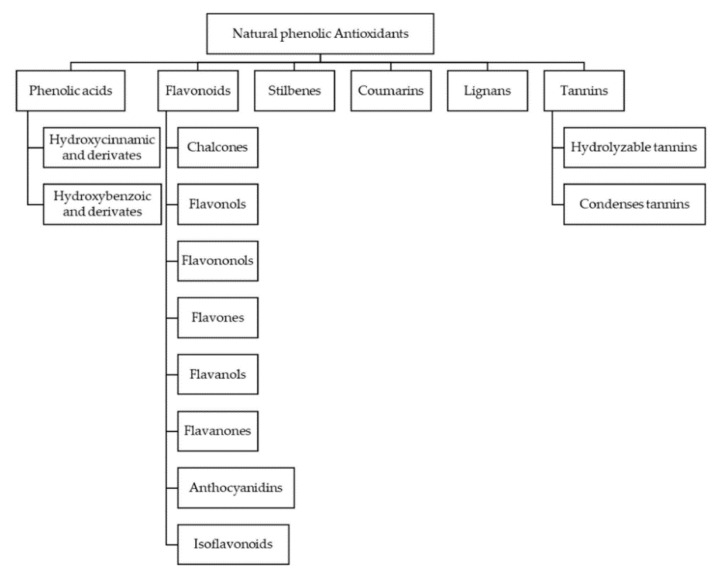
Natural phenolic compound classification [8].

The largest group within natural antioxidant compounds is the group of polyphenols, consisting of very diverse chemical compounds that can be classified in many ways, but that generally are divided into two main classes: flavonoids and non-flavonoids. Within the class of non-flavonoids, natural polyphenolic compounds can be present in chemical structures, such as: phenolic acids, stilbenes, lignans, chalcones, and tannins (hydrolysable and condensed) [34,35,36], Figure 1. Phenolic acids in wine act as copigments, and they do not impact odor and flavor. Stilbenes are the most well known as antioxidants, and within the chalcones group there is xanthohumol; present in beer, and of huge importance, as this compound possess high biological activity. Flavan-3-ols influence bitterness, astringency, and wine structure, and participate in the stabilization of color during aging. Tannins also contribute to the sensory characteristics, particularly of red wine, as they are related to the astringency, they also interact with other macromolecules (proteins and polysaccharides) influencing the colloidal behavior of wine. Condensed tannins (proanthocyanidins) in the brewing industry are interesting as they influence haze formation in beer. Anthocyanins are responsible for the color of red grapes and wines.

As is expected, due to different used raw material and technological processes, there are differences between wine and beer, in the presence, as well in the concentrations, of phenolic substances. Moreover, the antioxidant compounds in beer belong to different groups of chemical substances such are: thiols [37], SO_2_ [38] (product of the Maillard reaction [39,40]), α-acids derived from hops [41,42,43], and phenolic compounds [44,45,46]. Thiols have been suggested to correlate with sulfites in the antioxidative mechanism, and are important for beer’s oxidative stability. Sulfites were found to be the only compound that was able to delay the formation of radicals [38], and actually give antioxidant and antimicrobe properties in wine too. The main product of the Maillard reaction is melanoidin, which affects the color, flavor, and body of beer. Hop α-acids (also called humulones) represent the main bittering compound in beer, and have shown a high ability to quench radicals, while *iso*-α-acids possess this activity to a lower extent. In addition, *iso*-α-acids can influence beer staling, but not to a high degree. However, within this review, focus will be on the content of phenolic compounds, as they have been recognized as mainly responsible for antioxidant activity in wine and beer.

The polyphenol complexes of beer and red wine, additionally, represent a source of dietary antioxidants. As both beverages are very popular and widely consumed, benefits of the light-moderate consumption of wine and beer are supported by scientific literature data. Polyphenols from red wine and beer could act as antioxidants, and also as anti-inflammatory agents contributing to the defense against atherosclerotic pathologies [19,47]. The beneficial moderate consumption of beer is also based on antioxidant compounds present in beer, i.e., on their redox properties [48]. Antioxidants present in beer improve several diseases, and are associated with benefits to the cardiovascular and immunological system [19,20]. It was shown that after consumption of non-alcoholic beer, the decrease of several inflammation biomarkers, homocysteine, and systolic blood pressure occurred. These influences were mainly attributed to the polyphenolic compounds in beer. Furthermore, several studies have shown that the light-moderate intake of alcohol is associated with lower incidence of diabetes type-2, a higher level of high-density lipoprotein cholesterol, as well as with lipid oxidative stress reduction [49,50,51,52]. Torres et al. [53] reported that moderate wine intake, compared to other alcoholic beverages like vodka, rum, and brandy increased total antioxidant capacity, and decreased pro-inflammatory factors along with a fat-enriched diet that was consumed by young healthy volunteers. This is also supported by the phenomenon known as the “French paradox”, which indicates that moderate daily drinking of red wine contributes to lower coronary heart diseases incidence, despite their diets possessing a higher amount of saturated fatty acids and total fat [54]. However, excessive intake of alcohol beverages is associated with chronic disease development and other very serious problems, representing the leading risk factor for mortality [55]. Roercke et al. [3] reported there is an important influence of drinking patterns, such are episodic heavy drinking within average moderate drinkers, and some other important influencing parameters in term of health issues like smoking status, age, body mass index, and physical activity, and all of them have to be considered in order to estimate dose of alcohol as well the risks. After all, chronic heavy and episodic drinking should be avoided. In the Dietary Guidelines for Americans (2015–2020), moderate intake of alcohol proposes up to one unit of alcohol per day for women and two for men [56].

However, the positive influence of single polyphenols on human health occurs at higher concentrations than those found in beer and wine, indicating the synergistic action of different polyphenolic mixtures [57]. Ranges of some of the most important phenolic compounds, found in red wine and beer are presented in Table 1. Phenolic acids also possess antioxidant and anti-inflammatory properties [58]. Based on literature data, beer has shown higher upper values for content of *p*-coumaric acid, and all hydroxybenzoic acids, while for other polyphenolic compounds it was mainly the opposite, and higher concentrations dominated in red wine. Flavonols are considered very important bioactive compounds, and have shown positive effects against certain cancers and cardiovascular diseases in some epidemiological studies [59,60]. Concentrations of all three presented flavonols (quercetin, myricetin, and kaempferol) were much higher in red wine than in beer. Stilbenes, particularly resveratrol, are the most associated with wine’s beneficial properties. Resveratrol is recognized as an antioxidant, anticancer, cardioprotective, and anti-inflammatory agent. Due to its bioactivity, trans-resveratrol was proposed for many diseases as a therapeutic agent [61]. The content of stilbenes is not comparable for wine and beer, as based on literature data these compounds are rarely, or never, found in beer. It was also indicated that flavan-3-ols may show cardioprotective activity, and their antioxidant activity was shown in some studies [59]. Flavones are also recognized as molecules with important biological activity (anti-tumor, antioxidant, and anti-inflammatory), and were used as treatment for some neurodegenerative disorders and coronary heart diseases [62]. Flavanones also belong to antioxidant agents and the found concentrations in wine and beer were very low, while a lower content of naringerin was found in red wine compared to beer. Tannins also showed potent radical scavenging, anti-inflammatory, and antioxidant activity [63], and much higher levels of condensed tannins were found in red wine. Besides the presented polyphenolic compounds there are also some compounds found in wine and not in beer and the opposite. Within the compounds found in beer, two very important ones are xanthohumol and melanoidin. Both, xanthohumol and melanoidin have shown antimicrobial properties, melanoidin also possess antihypertensive, prebiotic, and antiallergenic properties [64], while xanthohumol showed anti-cancer, anti-inflammatory, anti-obesity, etc. properties [65,66]. Depending on the raw material and brewing process, the content of melanoidin ranges from 0.58 mg/L in alcohol free beer to 1.49 mg/L for dark beer, while in blond beer 0.61 mg/L was determined [67,68]. In wine, among the phenolic compounds with biological activity, there are also anthocyanins. The most common anthocyanins found in red wine, malvidin-3-glucoside and malvidin-3-galactoside, have shown anti-inflammatory effects, and their synergistic activity was observed [69].

### 2.1. Analytical Methods for Determination of Antioxidant Activity in Beer and Wine

It is of huge value to mention the importance of the analytical methods applied for determination of antioxidant activity in these beverages. Antioxidant power in different functional food, as well the isolated antioxidant substances, were dependent on applied assays used for their determination, which is also confirmed by Di Pietro and Bamworth [7]. Antioxidant activity in beer has been mainly determined using electron spin resonance spectroscopy, based on spin trapping of radicals that have been forming at 60 °C [38,41]. Differences in the antioxidant activity of examined wines and beers have been shown, and the superior behavior of beer was only demonstrated using the β-carotene bleaching method, while hydroxyl scavenging assay is not reliable for beer assessment, as compounds in beer react with thiobarbituric acid [7]. However, in research, comparing different beverages on an “as is” basis, and equalized according to the alcohol concentrations and the total polyphenol content, red wine performed the best for most used assays, when used samples were not equalized. Recently, Wannenmacher et al. [12] examined in their study two assays, one based on a mechanism consisted of electron transfer, such are ferric reducing antioxidant power (FRAP), and the other an oxygen radical absorbance capacity (ORAC) assay based on the transfer of hydrogen atoms, which scavenge peroxy radicals, in order to determine the antioxidant capacity of beer. Antioxidant value obtained using ORAC correlated positively with free amino nitrogen, total nitrogen, and *p*-coumaric acid, while values obtained using FRAP correlated positively with total anthocyanogens, total polyphenols, and catechin content [12]. Furthermore, both assays correlated significantly with the sum of phenolic compounds in the examined beers.

In winemaking, the most commonly used methods for determination of antioxidant activity and total polyphenol content in wine are spectrophotometric methods. Spectrophotometry is used for monitoring the decrease in absorbance that occurs when a present antioxidant scavenges the added radical in the sample [101]. For these purposes different radicals have been used in order to determine antioxidant capacity in beverages, and among them the most frequently used are DPPH (2,2-diphenyl-1-picrylhydrazyl) [102] and ABTS (2,20-azino-bis(3-ethylbenzothiazoline-6-sulphonic acid) [103]. Moreover, with spectrophotometry, it is possible to monitor the reducing activity of phenolic compounds in wine using FRAP, a method based on regeneration of ferric iron to ferric(II) by phenolic compounds, and using the CUPRAC method based on regeneration of copper(II) to copper (I) ion [104]. Even though these methods remain a classic tool for the evaluation of antioxidant activity and phenolic content, there are also some electrochemical techniques used for determination of antioxidant capacity of wines, and the most used are differential pulse voltammetry on glassy carbon working electrode [105] and cyclic voltammetry [106]. Recently, Ricci et al. [107] examined analytical approaches, using a flow injection system with a sequential diode array and electrochemical amperometric detectors. They concluded that flow injection, coupled with a diode array and electrochemical amperometric detectors, is useful and can be successfully applied for measurement of antioxidant capacity and the total phenolic content in wines. Minkova et al. [108] compared the antiradical capacity of Bulgarian red wines, using two analytical methods (spectrophotometric and chemiluminescent) and showed that chemiluminescent assays were more efficient in the elimination of hypochlorite, compared to the superoxide anion, for all wine samples. García-Guzmán et al. [109] evaluated the polyphenol index in wine and beer samples using a tyrosinase-based amperometric biosensor, obtained via a novel sinusoidal current method, and good analytical performance of this biosensor was achieved in terms of stability, reproducibility, limits of quantification and detection, linear response range, and accuracy, using caffeic acid as a polyphenol reference. However, electroanalytical techniques proved to be an appropriate alternative, considering that they showed quick response and good sensitivity, without sample treatment, simple instrumentation, low cost, etc. In addition, combined use of electroanalytical techniques and enzymes provided good selectivity in determination of polyphenol index [109].

Overall, there are contradictory and insufficient data on the correlation between antioxidant activity and the concentration of individual and total polyphenols in wine [32]. These contrasting data are due to differences in the used raw material, and also due to the proportions and contents of particular phenolic compounds. Nevertheless, it is not easy to compare the observed literature data, and sometimes it is even not possible, due to differences in applied methods, based on different methodological approaches. In Table 2 are presented ranges of total content of polyphenolic compounds and some phenolic classes, as well antioxidant activity measured with different analytical tools. Comparing obtained data, when the same methods were used, it was observed that wine dominated in the content of total polyphenols (FC method) and antioxidant activity (FRAP method), as was expected. However, it should not be forgotten that there are many influencing parameters, due to the different proportions of certain phenolic compounds in wine and beer, as well as due to differences in the used grape varieties in wines, and the type of beer and brewing technology.

### 2.2. Non-Flavonoid Polyphenols in Wine and Beer

**Figure 2:**
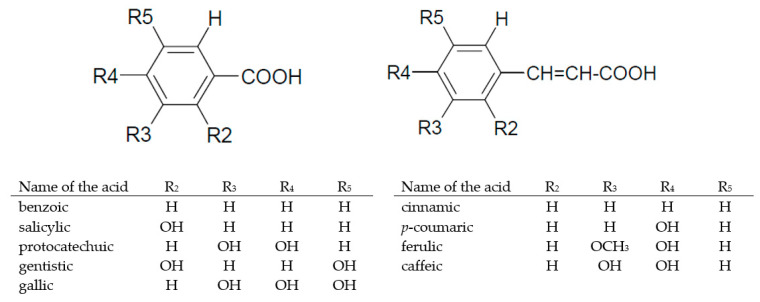
Hydroxybenzoic and hydroxycinnamic acids derivates [74].

Non-flavonoid phenolic compounds of wine consist of three main groups: two types of phenolic acids (cinnamic and benzoic), and stilbenes [95]. Beer also contains the non-flavonoid polyphenols, and within this group there are present the monophenolic compounds, chalcone (xanthohumol) and resveratrol [11]. Phenolic acids in both beverages can be found in the form of (hydroxy-) benzoic and (hydroxy-) cinnamic acids derivates, and after are classified according to the nature and the type of their ring substituent [36], Figure 2. The greater part of the determined phenolic acids in beer were found in bound form as esters, glycosides, and bound complexes. In wine, these phenolic acids can also be found as esters with tartaric acid, as well in the free form, or esterified with anthocyanins and ethanol. Additionally, in wine hydroxybenzoic and hydroxycinnamic acids act as copigments.

#### 2.2.1. Hydroxycinnamic Acids

Hydroxycinnamic acids in both beverages do not impact the odor and flavor, but they are very important compounds, as they act as precursors for volatile phenolic compounds [9]. In regards to beer the majority of studies reported that ferulic and *p*-coumaric acids are the most abundant acids in beer [45,46,73,74,75,76,77,84]. Hydroxycinnamic acids originate from the raw material used for beer production, i.e., barley malt and hops. Moreover, ferulic and *p*-coumaric predominate in barley malt, and their concentrations are influenced by the malting process, and growing environmental conditions, as well as the post-harvest treatment of barley. It was shown that ferulic acid is one of the most abundant acids in Chinese beers [77,114], European beers [74], and in Chilean beers [115]. However, published values for these two acids vary widely due to the differences in used extraction and analytical methods, as well as due to the form of the analyzed acids (free or bound) [116]. The content of phenolic acids was quantified by Floridi et al. [78], and in 23 Italian lager wines, *p*-coumaric acid was found at 1.364 ± 0.709 mg/L, while ferulic content was 2.41 ± 0.875 mg/L. Recently, Habschied et al. [9] quantified the content of caffeic and *p*-coumaric acids in different types of industrially produced beers (black, dark, lager, and pilsner) and noted that the content of caffeic acid was the lowest among all determined phenolic compounds. They also found that light and dark beers had the lowest share of *p*-coumaric acid, while black beers contained higher concentrations of *p*-coumaric acid. It was also shown that in the ale type of beer, the highest share among these acids belongs to caffeic acid [79], while ferulic acid was dominant in black, non-alcoholic, wheat, abbey, bock, and pilsen beers [46]. It is also important to note that hydroxycinnamates in beer can also be found in conjugation with the polyamides hydroxycinnamic acids amides or phenolamides, and after these compounds can be found glycosylated, and as derivatives of hydroxylated agmatine. These compounds, hordatines, and hydroxycinnamoyl agmatines have been detected in final beers, contributing to the beer’s astringency. The content of hordatine in final beers (determined as *p*-coumaric acid equivalent), in large number of samples ranged 5.6 ± 3.1 mg/L, showing that this substance group is the most abundant phenolic substance in beer [117].

Hydroxycinnamic acids can be found in their free form, or in bound form as tartaric esters. These acids represent the majority of the nonflavonoid class in red wines, and the majority of phenolics in white wines. These esters can be partially hydrolyzed during the process of alcoholic fermentation, resulting in their free forms. In grape and wine, caffeic, coumaric, and ferulic acids are also the most important in this sub-class of polyphenols [118]. Content of these acids in grapes, grape juice, and wine depends on the grape variety, as well on the environmental growth conditions. These compounds depend on variety; the influence of the vintage is not negligible, as well as the used winemaking technology. However, the content of caffeic acid was found to be the most abundant [70,71,119]. These data are not in accordance with results obtained by Lima et al. [72], in whose study the predominant acid was *p*-coumaric (2.30–6.70 mg/L), followed by caffeic acid (0.52–1.49 mg/L), while the content of ferulic acid was up to 0.16 mg/L, in wines from the most used grape varieties in Portugal.

#### 2.2.2. Hydroxybenzoic Acids

Hydroxybenzoic acids possess a general C6-C1 structure and belong to the phenolic compounds, Figure 2. In beer the most abundant hydroxybenzoic acids are salicylic, *p*-hydroxybenzoic, vanillic, and gallic acids, while in wine there are *p*-hydroxybenzoic acid, syringic acid, vanillic acid, and gallic acid. In regard to the contents of these acids in beer, Floridi et al. [78] found 2.866 ± 1.553 mg/L of salycilic acid, and 16.84 ± 10.988 mg/L of *p*-hydroxybenzoic acid in research on 23 Italian lager beers. Vanillic acid in beer varied from 0.08 to 2.98 mg/L [45,73,75,77,84], and in research from McMurrough et al. [76] varied between 2.5 to 12.7 mg/L, while in the same research gallic acid ranged from 1.1 to 3.5 mg/L, and in study by Zhao et al. [77] this acid valued from 1.81 to 10.39 mg/L. Recently, Wannenmacher et al. [12] determined the content (0.15–0.33 mg/L) of *p*-hydroxybenzoic and (0.07–0.22 mg/L) of protocatechuic acid in beers by varying the type of raw material and brewing technology. Special attention was put on research of gallic acid content in beer, as it was shown that this acid can be an indicator of oxidation during production of beer, due to its high susceptibility to degradation and oxidation [83]. It was shown that gallic acid predominates in Serbian and Brazilian beers [120,121], and in regard to the type of beer, this acid was found to be highest in lager beers [77]. However, recently, Habschied et al. [9] determined the highest concentration of this acid in black beer (14.22 mg/100 mL), and the lowest concentration in light style lager beer (4.12 mg/100 mL). These results aligned with results reported by Zhao et al. [78], while Mitić et al. [120] reported lower concentrations of gallic acid in bar beers, but still it was the major polyphenolic compounds in all samples. As in beer, gallic acid is also considered as the most important acid in wine, considering that this compound is the precursor of all hydrolysable tannins. The origin of gallic acid could therefore be from the hydrolysis of condensed tannin and gallate esters of hydrolysable tannins [122]. Furthermore, because of its three free hydroxyl groups this acid is considered as a very potent antioxidant, and higher concentrations of this acid using longer maceration times in winemaking of reds were obtained. The total amount of hydroxybenzoic acids in wine was found to be up to 218 mg/L [6].

#### 2.2.3. Stilbenes

**Figure 3:**
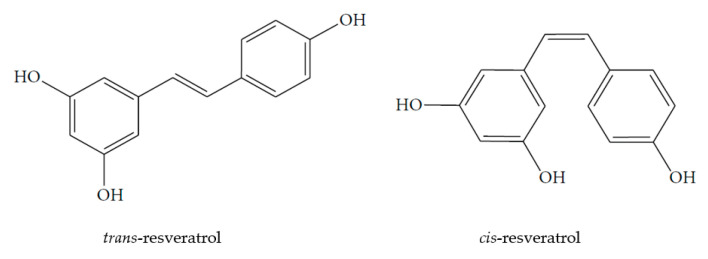
Structure of *trans*- and *cis*-resveratrol [135].

One of the most important compounds from the non-flavonoid class, which has received attention thanks to its link to beneficial effects on human health, is resveratrol [123,124]. Many researchers reported the health benefits of this compound, and its ability to prevent a number of human diseases [17,125,126,127,128,129]. In regard to its structure, resveratrol possesses two phenol rings connected by the styrene double bond [130], and it can exist in *cis-* and *trans*-configurations, Figure 3. Resveratrol can also be found in the form of 3-*β*-glucoside, *trans-,* and *cis*-piceid. Reported values of this compound were much lower in beer in comparison to wine, particularly red wine. Both isomers, *trans-* (0.7–6.5 mg/L) and *cis*- (0.1–7 mg/L) were detected in wine [87,88], and their concentrations depended on the used grape variety, terroir, applied viticulture practices, and the type of wine [131]. The content of *trans*-resveratrol was determined in wines from the Montenegrin terroir, and it was shown that contents varied from 0.62 ± 0.02 mg/L in Cabernet Sauvignon, to 1.27 ± 0.11 mg/L in Vranac wine [70]. Additionally, in this research, the content of *cis*-resveratrol in wines was 0.47 ± 0.03 mg/L in Vranac, and 0.57 ± 0.03 mg/L in Kratošija wine, while lower concentrations were observed in Cabernet Sauvignon wines (0.25 ± 0.01 mg/L). Similar results were obtained by Zoechling et al. [89] and Pajović et al. [71]. The concentrations of resveratrol ranged from 1.99 and 81.22 μg/L in 110 commercial beers [86], while these values for red wine were reported from 2.03 to 11.7 mg/L [86]. However, resveratrol has also been found to a lesser extent in alcohol-free and lager beers [132], and in the ale type [133]. This can be explained by the fact that resveratrol is found in hops, and a low amount of hop is in generally added during beer production, particularly when using the classical hoping regime [134].

#### 2.2.4. Hydrolysable Tannins

Tannins belong to a very important subgroup of polyphenol compounds, especially in red wine, as they contribute to the sensory characteristics of wine related to the perception of astringency and are also involved in reactions that lead to wine browning, particularly in white wine [6]. According to chemical structure they are divided into to two main classes, i.e., condensed tannins (proanthocyanidins) and hydrolysable tannins. Condensed tannins are polymers of flavan-3-ol, which classifies them into the flavonoid type of phenolic compounds, which will be described more later; they are also present in grapes, and after in wine. Hydrolysable tannins are a natural part of oak barrels, and can be found in wine matured in oak barrels. In red wine, the total concentrations of tannins determined in red wine vary from 1.1 to 3.4 g/L [91,136].

**Figure 4:**
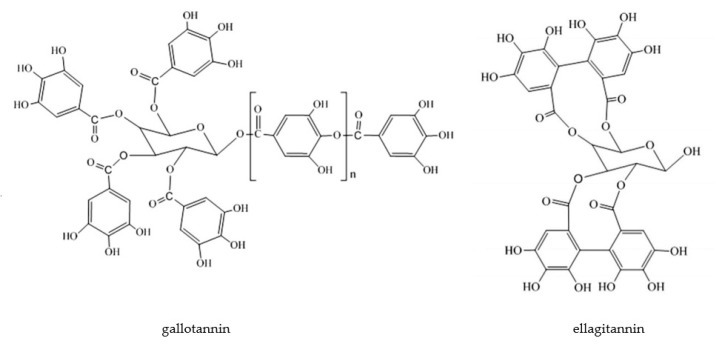
Structure of hydrolysable tannins, gallotanin, and ellagitannin.

The precursor and basic unit of hydrolysable tannins is gallic and its derivatives, i.e., ellagic acid, and these acids are mainly esterified with sugars, such as glucose, or less commonly quinic or shikimic acid, Figure 4. Due to this esterification they can achieve from 500 to 2800 Da. They are very influenced by pH changes, through which they can be degraded, if some enzymatic or non-enzymatic processes occur. Hydrolysable tannins are usually extracted from oak barrels during wine maturation, therefore aging in oak mainly promotes extraction of ellagitannins into the wine. Depending on the type of the wood used for wine maturation, concentrations of hydrolysable tannins range from 0.40–50 mg/L [90,91,92,93,94]. Hydrolysable tannins in beer originate from hop and malt [11]. Marova et al. [80] determined the concentrations of hydrolysable tannins in 22 samples of commercial beer in amounts of 1.5 mg/L of (+)-catechin gallate and (−)-epicatechin gallate.

### 2.3. Flavonoid Compounds

Flavonoids represent the largest group belonging to the polyphenolic compounds, and in wine 85% of the phenolic compounds are accounted for by the flavonoids [137]. The basic structure of flavonoids consists of a system with three-rings: two aromatic and one oxygen-containing central ring [138]. Based on the substitution of the pyran ring and on its oxidation degree, flavonoids are classified into a wide range of subgroups, such as flavones, flavonols, flavanes, flavanols, flavanones, flavononols, anthocyanins, and anthocyanidins, as well as the chalcones and dihydrochalcones [139]. Both beer and wine contain the flavonols, flavanols, anthocyanins etc., but there is one important compound present in beer not found in wine, which is found only in hops, and that is xanthohumol and its cyclization product-flavanone, isoxanthohumol, both of which have shown anti-cancer properties [140,141]. Xanthohumol belongs to the group of prenylated chalcones, and this compound has been widely researched due to its biological activity [142,143]. Its biological and technological aspects, as well the chemistry of this compound, have been deeply reviewed [142]. Concentrations of xanthohumol depend on brewing technology and hopping regime, and the levels of this compound in commercial beer were around 0.2 mg/L [144]. These prenylflavonoids also turned out to be important for beer aromas and flavor, particularly in dark beers [80,83,145], and they are chemically related to the bitter hop acids (which are also biologically active, particularly α-acids) and polyphenols. Beer is considered as the main source of this molecule, with concentrations varied, from 0.002 and 0.628 mg/L [80].

#### 2.3.1. Flavan-3-ols and Condensed Tannins

**Figure 5:**
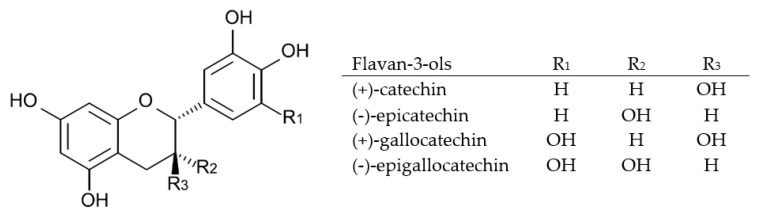
Structure of flavan-3-ols.

Flavanols are basically benzopyrans, and these compounds can be found in the form of simple monomers as well in polymers, Figure 5. The best known and important molecules within this group are catechin and its enantiomer, epicatechin. They represent the precursors for the formation of proanthocyanidins that give the structure and astringency to beer and wine [9,137]. Moreover, wine is considered as the beverage with higher concentrations of these compounds (50–120 mg/L) [96,97,146], while in beer the concentrations varied 1–20 mg/L [147]. In some special, and very old, red wines, the content of catechin was noticed even up to 1000 mg/L [148]. Catechin and epicatechin can be found in the grape stems, seeds, and skin, and after in wine [95], while beer catechins are also derived from raw material used for beer production, i.e., from barley/malt and from hop [149]. The flavan-3-ols and their contents were evaluated by many authors [73,74,75,78,80,81,82,100,150], and among these compounds, catechin was the one most described and abundant in beer [74,81,82,150]. Besides monomers, in beer also can be found their esters with gallic acids (catechin gallate), and catechin derivatives (epigallocatechin, gallocatechin, epigallocatechin gallate, and epicatechin gallate) were also determined in grape and wines. Recently, Wannenmacher et al. [12] determined the content of (+)-catechin (2.74–6.54 mg/L) in beers made with different technological variations.

**Figure 6:**
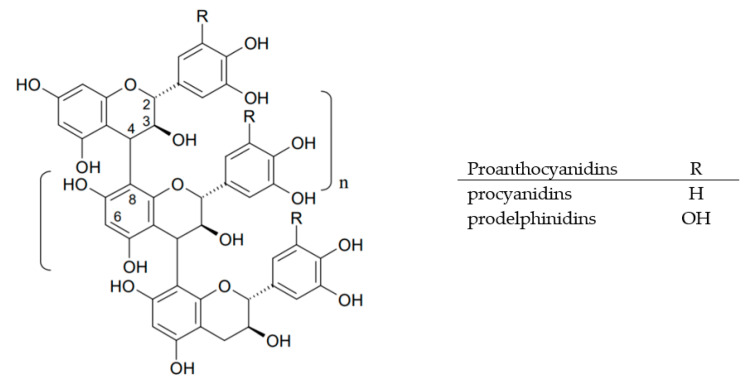
Structure of proanthocyanidins.

Proanthocyanidins are phenolic compounds, and structurally represent oligomers flavan-3-ols, also known as condensed tannins, or in the brewing industry as “anthocyanogens” [12]. These polymeric compounds, transformed to anthocyanidins, can be found in all parts of the grape berry (pulp, skin, and seeds) and are transferred into wine during grape processing (crushing) and during alcoholic fermentation and maceration [151]. The structure of proanthocyanidin depends on the degree of polymerization, hydroxylation pattern and the stereochemistry, the type of the connection between monomers, and the 3-hydroxyl group esterification. Dixon et al. [152] deeply reviewed this type of phenolic compound, and according to the nature of their monomers proanthocyanidins were classified into propelargonidins, prodelphinidins, and procyanidins, Figure 6. Monomers of epicatechin and catechin make up the procyanidins, while epigallocatechin and gallocatechin subunits make up the prodelphinidins, while the propelargonidins consist of mixed oligomers, containing at least one monomer with a 4′-monohydroxyl group. In the brewing industry, proanthocyanidins became interesting due to their relation with the haze formation in beer, and it was shown that the haze in beer increases with higher molecular weight. In addition, proanthocyanidins also became the focus of researchers due to their highly potent antioxidant capacity and possible positive effects on human health, particularly in cardiovascular diseases and cancers [153,154]. Prodelphinidins and procyanidins have been detected in beer and wine as well [95,149]. These two groups in wine lead to delphinidin and cyanidin, and represent the most abundant condensed tannins in grape and wine. Gu et al. [155] determined the proanthocyanidins concentration in beer as 23 mg/L, that is 23-fold lower compared to grape juice, and around 13-fold lower compared to red wine. The main part of proanthocyanidins in beer consist of dimers (11 mg/L), monomers (4 g/L), tetra- to hexamers (4 mg/L), and trimers (3 mg/L), while the main part of proanthocyanidins consist of 37% of tetra- to decamers, 35% of >10-mers, and 28% of mono- to trimers [155]. Proanthocyanidins are very important for wine sensory characteristics as they influence the astringency and bitterness of wine, and also play an important role in the process of wine maturation and aging [95]. The level of astringency and bitterness is affected by the molecular size of the proanthocyanidins, and it was proposed that bitterness comes more from the monomers, while the astringency from the larger molecules [156].

#### 2.3.2. Flavones, Flavonols, and Flavanones

**Figure 7:**
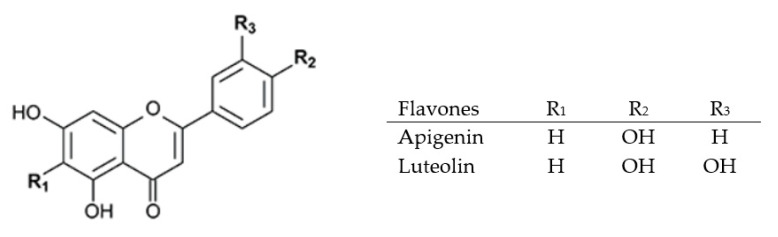
Structure of flavones [158].

The 4-keto group is the shared characteristic of this group of phenolic compounds. Flavones contain three functional groups, the carbonyl and hydroxyl groups, and a double bond within the flavonoid skeleton, Figure 7 [6]. Flavones were determined in grapes, i.e., in skin and in wine in two forms: aglycones (nonsugars) and glycosides. They were not found in significant levels in grapes and wine, except for luteolin, where concentrations in grape ranged from 0.2 to 1 mg/L [95,96,97,98]. It is known that flavones possess important biological characteristics, which are beneficial for human health, including anti-tumor, anti-inflammatory, and antioxidant features [62]. Gerhäuser et al. [157] determined flavones that were isolated in aglycon form from un-stabilized beer, such are apigenin, tricin, and chrysoeriol, and in glucoside form, i.e., apigenin derivatives. It has been shown that the most likely origin of these derivatives is from barley. Kellner et al. [82] determined in commercial beers the content of apigenin, from 0.80 to 0.81 mg/L, while Marova et al. [80] found 0.10 to 0.19 mg/L of luteolin in commercial beers. Apart from flavones, beer also contains the isomer of this phenolic group, i.e., isoflavones, and within them belong daidzein, genistein, formononetin, and biochanin A [82].

**Figure 8:**
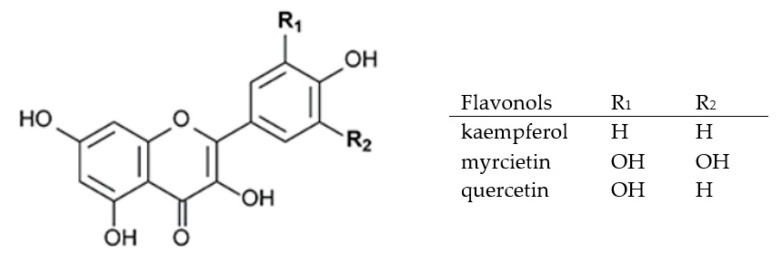
Structure of flavonols [158]. Reproduced with permission from [Drake V.J.], [Linus Pauling Institute]; published by [Oregon State University], 2005.

The characteristic functional group for flavonols is a hydroxyl group attached to C3, and because of that they are often named as 3-hydroxyflavones, Figure 8. In red wine are found aglycon forms of flavonols, such are kaempferol, quercetin, rutin, and myricetin, as well as their glycosides, which can be found as galactosides, glucuronides, glucosides, and diglycosides. Concentration of these flavonols was found in wine at levels from 12.7 to 130 mg/L [96,97,98]. The biological activity of these compounds has been described, particularly as improving cardiovascular health [159]. Speaking about beer raw material, it has been shown that flavonols occur less in cereals, and two of them in aglycon form and their glycosides (quercetin and kaempferol) have been found in hops. Moreover, kaempferol and quercetin glycosides, as well as their malonyl esters, have been determined in hop [160]. Recently, Gangopadhyay et al. [161] detected quercetin in barley flour in the content of 15.1 μg/g dry weight. Relatively high concentrations of quercetin were measured in lager beers (1.72–1.79 mg/L) [74], and a high concentration of kaempferol (1.64 mg/L) was measured in beer [100]. Comparing this literature data, it is obvious that wine is richer in content of this phenolic group.

**Figure 9:**
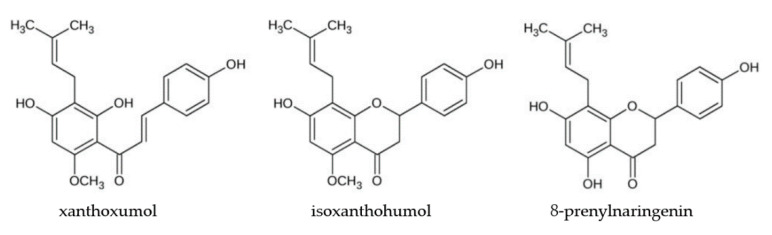
Structure of xanthohumol, isoxanthohumol and 8-prenylnaringenin [163].

When it comes to the flavanones, beer is much more interesting, as it contains four prenylflavanones, isoxanthohumol (the most abundant one) in concentrations from 0.04 to 3.44 mg/L [162], and then there are 6- and 8-prenylnaringenin and 6-geranylnaringenin [141], Figure 9. Isoxanthohumol is formed during the brewing process by isomerization of xanthohumol. Marova et al. [80] determined the content of naringenin in 22 commercial beers, and it varied from 0.06 to 2.34 mg/L, while the total naringenin content in four German red wines varied from 0.9 to 4.2 mg/L [89].

#### 2.3.3. Anthocyanins

**Figure 10:**
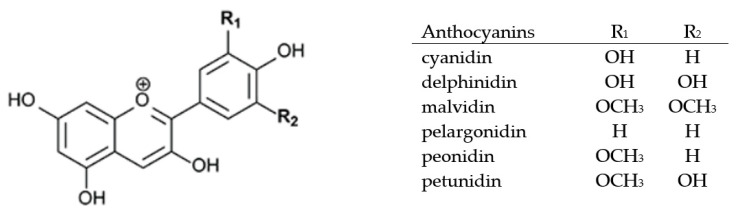
Structure of anthocyanins [158].

When it comes to the anthocyanins, according to the literature survey this phenolic group of compounds were extensively reported in wine, while there were no reports of anthocyanin content in beer. Anthocyanins are proved to be promising agents against some diseases [6], and there are many reports on their protective role against coronary heart disease [164,165,166,167]. Different anthocyanin derivatives were determined in grapes and wine, as well as in the medium similar to the wine [95]. In regard to the chemical structure of anthocyanins, anthocyanins represent the glycosylated form of the anthocyanidins, and both contain as a nucleus the flavylium (the 2-phenylbenzopyrylium) cation, with methoxyl and hydroxyl groups attached to the different positions [6], Figure 10. Anthocyanins are principally responsible for the grape and wine color, and there are six anthocyanins detected, namely cyanidin, malvidin, peonidin, petunidin, delphinidin, and pelargonidin in red grapes and wines [6], as well as their 3-*O*-monoglucosides [151,168]. Anthocyanins are mainly found in the grape skin, and malvidin is one of the highest representatives in *Vitis vinifera*. The content of anthocyanins is influenced by the grape variety, i.e., it is relatively stable for each grape variety, and concentrations can vary between the vintages due to environmental conditions, i.e., clime conditions and terroir, also their concentration depends on the winemaking process, particularly during maceration, i.e., extraction that occurs during alcoholic fermentation. In addition, anthocyanins are involved in important reactions, such are polymerization, oxidation, and formation of new pigments during the process of winemaking and wine maturation. The total content of anthocyanins in wine can widely range depending on grape variety, from 32.5 to 1011 mg/L [71,111,112,119].

## 3. Impact of Technologies in Order to Increase Phenolics in Wine and Beer

**Scheme 1:**
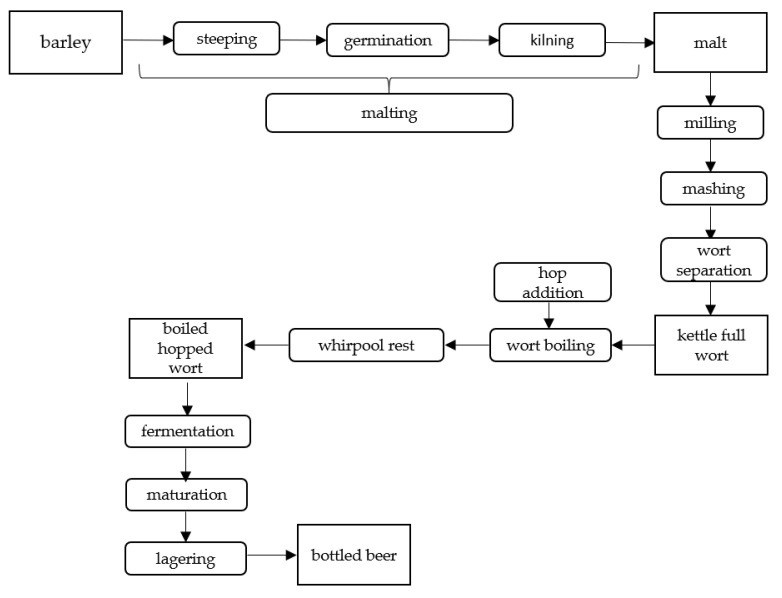
Basic steps in brewing technology.

**Scheme 2:**
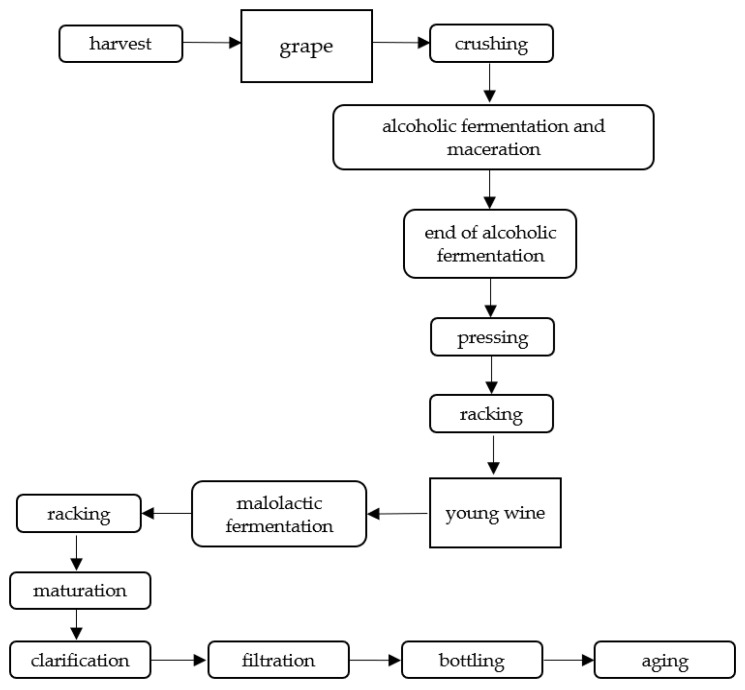
Basic steps in winemaking technology.

The only material for wine production is grape, while for beer production there is malt (sometimes along with some adjuncts such as rice, sugar, corn, and wheat), water, and hop. The process within both the production of beer and wine is alcoholic fermentation and, for that purpose, usually commercial dry yeasts are used. In this section, through the processes of making wine and beer, will be highlighted possible methods used for improving the phenolic content in these two beverages. Basic brewing and winemaking technology are presented at Scheme 1 and Scheme 2, respectively.

### 3.1. Raw Material

As was mentioned, phenolic content of grapes depends on grape variety, terroir (clime and vineyard location, soil type), applied vinicultural agro-techniques, harvest date, applied oenological practices, and wine maturation. Nowadays, in regard to grape growing, it is very demanding and challenging to produce healthy grapes, with the optimal level of maturity (phenological and technological), and not over-ripened, due to climate changes with which the world today is faced. Climate changes significantly influence grape ripening, for example in hot and dry vintages the process of grape ripening is very fast, giving the grapes a non-balanced maturity with high sugar content, but with the lack of phenolic compounds, which results in wines with astringent and green tannins [169,170]. In order to deal with this issue, some novel agricultural practices have been investigated. Some of them are cluster thinning and defoliation [171,172,173,174], which have been shown to have a beneficial influence on the synthesis particularly of anthocyanins and flavonoids. Additionally, the influence of reduced yield on total phenolic content was observed [175], showing that there is no strong correlation among yield and the content of total polyphenols and anthocyanins, similarly to the antioxidant potential. Cluster thinning and early leaf removal showed an increase of proanthocyanidin and anthocyanin levels in wines of Cabernet Sauvignon and Vranac [171]. Besides these examined agro-techniques, there are also some reports that used elicitors in order to enhance the resveratrol content [176,177]. Recently, Giacosa et al. [178] investigated foliar application of *Saccharomyces cerevisiae* inactive dry yeasts, and concluded that the effect of vintage was very important, as in one year there were significant differences between treated and control grapes, while in another vintage treated wines obtained a lower phenolic compounds content. Therefore, this foliar application could be efficient in conditions that are critical for synthesis and thermal degradation of some phenolic groups, such are anthocyanins [178].

Two main beer ingredients from which phenolic compounds originate are cereal (mainly barley) and hop. Barley (*Hordeum vulgare* L.) is a member of the poaceae family, and for the purposes of malting and brewing, usually two-rowed spring barley varieties are used. Barley contributes from 70–80% of total polyphenolic compounds in beer, even if it was shown that barley malt possesses a lower content of total polyphenolic compounds than hops [179,180]. Barley contains various groups of phenolic compounds, which mainly consist of phenolic acids (free and bound form) Their total content varies from 604 to 1346 μg total phenolic acids/g barley flour [181], lignans (3.7 μg/g of total content) [182]. Hordatines in concentrations from 72–178 μg/g dry weight were determined by Kohyama and Ono, [183] as well as flavanols in concentrations from 325 to 527 μg/g barley flour [181]. However, like in grape varieties, in barley the composition and concentration of the polyphenolic content is influenced by the variety of barley and place of origin. There are many reports on individual and total polyphenol content in regard to the different barley varieties [179,181,184,185,186,187,188]. Besides the variety of barley and growing conditions, polyphenolic content is also influenced by the type of barley; Holtekjølen et al. [181] showed that in hulled varieties the total phenolic content is significantly higher. As there is no research regarding increasing the phenolic content of the barley in the field, there are some reports on the phenolic changes due to variation of some technological brewing parameters [12,110]. Changes in regards to the phenolic content that occur during preparing malt and boiled hopped wort are deeply summarized in review by Wannemacher et al. [11]. The antioxidant activity of pale and dark beer was contributed to by malt [24], while antioxidant activity of beer was not influenced significantly by hopping [189]. Malt made of barley represents the most important starch used in the process of beer making. Production of malt consists of the following phases: steeping, germination, and kilning. During steeping there is an increase in water content, rootlets and sprouts develop during germination, and kilning represents drying of the mass. A decrease in phenolic content occurs during the steeping phase, due to the leaching of phenolic substances, but during germination and kilning the content of phenolic compounds increases [11]. Narziss [179] has shown that an increase of polyphenolic content occurs during malting, and highlighted the kilning step as the most important for solubilization of polyphenols. There are five phases during the kilning process: heating up (start up with establishing air flow), removal of free water (i.e., drying (temperature goes from 50 to 60 °C)), increasing air temperature (intermediate drying), bound water removal, and at the end curing, during which the moisture content of grain increases to 5 and 8% [190]. With variations of parameters during the process of malting, such as degree of steeping, and time and temperature of germination, it is possible to adjust the quality of malt. Muñoz-Insa et al. [191] have shown that there is a positive correlation between the germination temperature and the total phenolic content, and a negative one between higher degree of steeping and the total phenolic content. Recently, Wannemacher et al. [12] investigated the impact of malt modification and hopping regime on the antioxidant potential of beer, and concluded that malt with higher raw protein content gives a beer with significantly higher antioxidant activity, determined using ORAC assay.

Hops (*Humulus lupulus* L.) belong to the cannabaceae family and are added in small amounts to beer in order to provide the final aroma and bitterness, as well to impart antibiotic and antifungal properties [192]. Hop is rich with plenty of antioxidant compounds that can be resinous, like the prenylated chalcones and α-acids, or non-resin phenolic compounds, like flavonoids or phenolic acids [11,12,160,180,193]. There are four main valuable groups of ingredients found in hops: soft and hard resins, essential oils, and polyphenols [11]. Xanthohumol represents the main compound found within the hard hop resins, and is accompanied by 13 other compounds also belonging to the prenylated chalcones, but in up to 1–100 fold lower concentrations compared to xanthohumol [142]. It was also confirmed that xanthohumol can be found only in hops, while other prenylflavonoids can be found in some other plant families [142,143]. In regard to the polyphenols present in hop cones, there are flavanols (32–191 mg/100 g air dry hops), proanthocyanidins (91–599 mg/100 g air dry hops) [194], flavanol glycosides (quercetin: 0.092 mg/100 g, kaempferol 0,12 mg/100 g) [195], and phenolic acids (hydroxycinnamic acids 59–288 mg/100 g air dry hops, hydroxybenzoic acids: <1–10 mg/100 g air dry hops) [194]. Jerkovic and Collin [196], investigated the total content of *trans*-resveratrol and *trans*-piceid, which ranged between 0.5 to 11.7 mg/kg in 40 samples of hop cones. They also concluded that harvest year strongly influenced the content of stilbenoids in hops, as well as that hop varieties with a lower content of α-acid usually contain a higher content of stilbenoids [196,197]. It was found that the vintage, i.e., the harvest year and date of the harvest influence, to a large extent, the quantity and quality of polyphenols in hop cones. Inui et al. [198] found that the content of polyphenols increases if the harvest was performed earlier, but the development of specific polyphenolic compounds differed. Kavalier et al. [199] showed that the content of some terpenophenolics increased during hop ripening, but the tendency of flavanols, flavonols, and phenolic acids was not clearly defined. It was also determined that the content of proanthocyanidins in hops is influenced by the growing conditions as well as by the hop variety [200]. Besides the influence of hop variety and growing conditions, i.e., the harvest year, the content of polyphenols in hops also depends on the type of hop product [180,201]. Mainly, hop is processed into the hop extracts, hop pellets, and isomerized products. During production of hop pellets, the raw hop goes through processes such as drying and grinding, which cause slight loss of polyphenol content, while the process of pelletization did not significantly influenced the content of polyphenolic compounds [202]. It has also been shown that the time length of storage influences the concentration of polyphenols in hop, and Mikyška and Krofta [203] showed that after twelve months of storage the polyphenols content had decreased significantly. In regard to hop extracts it was shown that the type of polyphenolic compounds and their concentrations depend on extraction solvent. In this respect, hop extract obtained using supercritical CO_2_ is used as a source of xanthohumol, because flavanones and other prenylated chalcones are insoluble in this kind of solvent [204]. Quiet recently, production of hop polyphenol, as well as tannin extract, has been used in order to improve light stability and the storage of beer [205,206].

After malt production and prior alcoholic fermentation, the next step in beer making is wort production and its boiling with hop addition. During these brewing processes, changes of the total and individual polyphenol content occurs constantly. Therefore, all important influencing parameters should be considered. First, before malt undergone mashing, it has to be milled. There are two types of milling, dry, and wet with water addition. It was shown that the total phenolic concentration and ferulic acid content decreased during wet milling [207]. Mashing technology can be performed in two ways, one in which the whole mash is treated with heating steps that are aligned with the activity of enzymes, and another in which part of the mash is separated and heated in another kettle, and after this part has undergone boiling in order to inactivate enzymes it is returned to the main mash, at the same time increasing the temperature in the main tank. It has been shown that temperature, mashing-in time, the thickness of mash, as well the grist coarseness influence release of phenolic acids. Vanbeneden et al. [208] found that the optimal temperature of cinnamoyl esterase is 30 °C, while optimal temperature for ferulic acid release is 40 °C. Other researchers also confirmed that temperature of 40 to 45 °C is optimal for releasing ferulic acid [209,210]. Vanbeneden et al. [208] showed that more ferulic acid was released using finer grists, and longer mashing time gave wort with a higher level of ferulic acid [209]. Overall, it was shown that only small part of hydroxycinnamic acids had been transferred into wort during the mashing process, the majority was left in the consumed grain. Zhao et al. [211] found that the total phenolic content decreased during the phase of enzyme inactivation. After mashing, the following step is wort separation from the consumed grain, using a mash filter or a lauter tun, the special type of vessel for the purpose of filtering the spent grain. Higher reduction of the phenolic compounds content was found when lauter tun was used [212]. These finding are not in accordance with the results of Pascoe et al. [213], who found that during the lautering phase the concentration of total polyphenols increased, due to the extraction from phenol rich spelt material. After the wort is separated the next phase is its boiling and hop addition, and within these phases reaction of polymerization occurs, as well as precipitation and interactions between proteins and polyphenols. According to Forster and Gahr [214], the transfer rate of the total polyphenols from hop during boiling is from 50 to 70% and this rate is different for different groups of polyphenolic compounds, depending on their polarity as well as on their affinity to interact with the proteins from wort. Hop can be added at the beginning of wort boiling in order to obtain the desired bitterness, or at the end of wort boiling, or even during whirlpool rest, and in that way will influence final beer aroma. The whirlpool rest is the operation that follows wort boiling and hop addition, in order to get clear wort separated from hot trub, in which are left insoluble proteins, bitter and organic substances, and ash. During this process, a significant decrease of phenolic substances occurs due to their adsorption into the hot trub. Late hop addition was proved as useful for better oxidative beer stability [40,41]. Wietstock et al. [215] found that a modified dose of hop improved oxidative beer stability, and a lower content of staling aldehydes were determined after storage compared to single hop dosing at the beginning of wort boiling. Mikyška et al. [201] did not observe significant differences between the beer hopped at the beginning of wort boiling, with hop pellets (type 90), or hop CO_2_ extract. However, the early addition of hop and longer wort boiling resulted in a higher depletion of phenolic compounds [11,206]. Higher content of total polyphenols, un-isomerized α-acids, and anthocyanogens (flavan-3,4-diols) were achieved when a late hop addition regime was applied [40,41,44,45]. Wannemacher et al. [12] researched the impact of different hop products and hoping regimes on the concentration of total phenolic and antioxidant potential in beer and found that besides higher content of total polyphenols, the higher content of individual phenolic compounds was also observed in beer with second hop addition during whirlpool rest. Furthermore, in their research higher sensory scores were given to the beers in which hop was added during whirlpool rest. Recently, Mikyška [110] investigated the influence of different hopping regimes during wort boiling, and concluded that flavanols content (epicatechin, catechin) in hopped wort and beer mainly depends on the hop raw material, and in the case of the addition of an aromatic and polyphenols rich hop, that two thirds of the polyphenol level in beer is influenced by hops. They also found that the flavonols kaempferol, quercetin, and multifidol are present in wort and beer in their glycosidic form, and the origin of these compounds is solely from the hops. During wort boiling it takes 15–30 min to release the flavonoids from hop into wort, and the dose of hop and low-pressure boiling technology did not influence significantly the polyphenol content. The addition of hops with a higher content of polyphenols turned out to be a better source of these antioxidants, compared to the addition of hop extract in beer [110].

### 3.2. Changes during Alcoholic Fermentation

There have been more studies in regards to the changes of polyphenols content during alcoholic fermentation in wine comparing to the beer. The main reason for this is that during the alcoholic fermentation of grapes, particularly red, maceration, i.e., extraction of polyphenolic compounds from grapes, occurs. Beside the chosen grape variety, this process is affected by many microbiological and technological parameters, such as yeast, enzymes, temperature, and the applied vinification techniques. During alcoholic fermentation, polyphenolic compounds in wine increase, while the concentration of total phenolic substances during fermentation in beer decreases [212].

Brandolini et al. [216] have shown that the yeast strain used for alcoholic fermentation, besides having an important role in the sensory quality of wine, also influences the polyphenol content. In the research of Kostadinović et al. [217], it was shown that by using dissimilar yeast strains it was possible to influence the stilbenes concentrations and antioxidant activity in Merlot and Vranac wines. Similar results were obtained for the wines Albariño [218], Pinot Noir [219], and Gaglioppo [220]. Investigating singular yeast strains, confirmed that the used starters have the ability to influence polyphenolic composition of wine. The importance of inoculation with commercial yeast starters, in order to modulate the content of total polyphenols in wine, was also highlighted [111]. Recently, Grieco et al. [27] investigated the influence of autochthonous yeasts isolated from the grapes of varieties Primitivo and Negroamaro grown in the Apulia region, and they determined significantly higher total concentrations of stilbenes in wines of both varieties fermented with autochthonous yeast (18.40–67.78 mg/L) than with commercial ones (3.70–18.83 mg/L). In their research the sum of determined phenolic acids (caftaric, caffeic, and *p*-coumaric acid), in wines fermented using commercial yeast ranged from 350.90 to 677.80 mg/L, while in wines fermented with autochthonous yeast strains, it ranged from 731.00 to 1976.70 mg/L. Similarly, the sum of determined flavonols (myricetin, quercetin, and kaempferol) varied up to 106.1 mg/L in wines fermented with an autochthonous yeast strain, and up to 49.80 mg/L in wines obtained utilizing commercial yeast. Only the content of identified flavanols (catechin and epicatechin) did not show significant differences, varying from 16.07–20.09 mg/L and 17.05–22.50 mg/L for wines fermented with commercial and autochthonous yeast, respectively. Total phenolic content and antioxidant activity in wines that utilized native yeast strains, obtained up to 1569.3 ± 7.6 mgGAE/L and 96.4 ± 1.5 mmol TE/100 mL, while the highest value of total phenolic content of wines fermented with commercial yeast was 1221.9 ± 7.6 mgGAE/L, and for antioxidant activity that value was 76.60 ± 2.1 mmol TE/100 mL. Their results indicated that the use of native yeast can considerably affect the composition of polyphenolic compounds. The significant influence of different commercial yeasts on the concentration of total, and some individual, phenolic compounds resulted in an increased of total phenolic compounds, and particularly stilbenes [70]. Besides the use of yeast, i.e., performing the traditional fermentation or using commercial selected dry yeast or isolated native yeast, it is also possible to vary other parameters. It was determined that higher content of phenolic compounds was observed performing alcoholic fermentation in fermenters comparing to traditional vinification in PVC barrels, and the addition of grape tannins, enzyme, and oak chips increased the content of total polyphenols, total anthocyanins, and total flavan-3-ols [33]. Recently, Generalić Mekinić et al. [28] investigated the impact of two different commercial pectolytic enzymes, which were based on polygalacturonase, with the activity of 7500 and 7600 units/g, on the phenolics extraction during maceration as well on the antioxidant activity of the analyzed wines. In their research, the use of commercial pectolytic enzymes had a slightly negative influence on the content of total phenolic compounds and antioxidant features of the wine. In the control sample, without enzyme addition, the content of determined phenolic acids varied from 0.91 ± 0.05 mg/L for protocatechuic acid, to 28.63 ± 0.04 mg/L for gallic acid; the content of flavonoids varied from 1.27 ± 0.03 mg/L for quercetin, to 82.60 ± 0.18 mg/L for catechin; the resveratrol content was 0.70 ± 0.02 mg/L, and anthocyanins varied from 0.36 ± 0.01 mg/L (petunidin-3-(6-*O*-coumaryoyl)glucoside), to 50.49 ± 0.15 mg/L (malvidin-3-*O*-glucoside). While in wines treated with enzyme (7500 polygalacturonase units/g) the content of determined phenolic acids varied from 1.67 ± 0.02 mg/L for protocatechuic acid, to 65.45 ± 0.02 mg/L for gallic acid; the content of flavonoids varied from 2.18 ± 0.01 mg/L for quercetin, to 91.99 ± 0.10 mg/L for catechin; the resveratrol content was 0.51 ± 0.02 mg/L, and anthocyanins varied from 0.29 ± 0.00 mg/L (cyanidin-3-(6-*O*-coumaryoyl)glucoside), to 39.29 ± 0.10 mg/L (malvidin-3-*O*-glucoside). In wines treated with enzyme (7600 polygalacturonase units/g) the content of determined phenolic acids varied from 1.78 ± 0.02 mg/L for protocatechuic acid, to 45.04 ± 0.19 mg/L for gallic acid; the content of flavonoids varied from 5.19 ± 0.12 mg/L for quercetin, to 55.49 ± 3.93 mg/L for catechin; the resveratrol content was 1.07 ± 0.13 mg/L, and anthocyanins varied from 0.21 ± 0.00 mg/L (cyanidin-3-(6-*O*-coumaryoyl)glucoside), to 21.63 ± 0.09 mg/L (malvidin-3-*O*-glucoside). Lower content of total phenolic compounds, particularly of monomeric anthocyanins in wines treated with enzymes, was probably caused by polymerization reactions, or due to glycosidase activity causing hydrolysis of these compounds [28]. Furthermore, the time of maceration also influenced the anthocyanin content, as well the wine color; low anthocyanin content and weak color was obtained in wines with a short period of maceration, while the prolonged maceration time resulted in unstable and poor color wine characteristics [221]. There are many studies regarding the use of different winemaking techniques and enzymes, and the obtained results are contradictory, but they all improve the knowledge, with the aim of choosing appropriate vinification techniques [28,221,222,223,224,225].

As was mentioned, knowledge in regard to the phenolic compound change during alcoholic fermentation in beer is incomplete, and the influence of different yeast strains and technological variations should be considered. However, by now the decrease of phenolic compounds during fermentation, warm rest, and chill-lagering is confirmed [212]. It was also found that these processing phases did not have a significant impact on the catechin and phenolic acids, except for ferulic acid, whose concentration decreased by 35% during warm rest at the end of fermentation [213]. Contrarily, Coghe et al. [209], noticed increase of ferulic acid during fermentation, which they attributed to the activity of enzyme in yeast feruloyl esterase.

### 3.3. Maturation, Aging, and Storage

Maturation and aging represent very important steps in wine and beer production. Changes in wine during the process of maturation and aging reflect, first, on the wine color and its sensory properties, in terms of the harmonizing astringency. Color change is associated with a decrease in anthocyanins content in aged wines, and changes depend on wine pH and SO_2_ content. Degradation reactions of grape-derived anthocyanins occur in an oxygen excess, forming the insoluble complexes of brown compounds [226]. Additionally, during wine aging, anthocyanins bind covalently with other compounds in wine, such as flavan-3-ols, then they form pyranoanthocyanin, and undergo the polymerization reactions; all these new compounds improve the wine stability, and there is less bleaching in the presence of SO_2_ [227,228]. The structure of formed compounds varies according to the molecular weight, from low flavanyl-vinylpyranoanthocyanins [229], and pyranoanthocyanins [230], and to the large molecules like tannin–anthocyanin adducts [231]. As one of the important factors also influencing the change of anthocyanin loss is the temperature of storage [231]. The choice of vessel for maturation and the aging time, beside the wine style, also influence sensory characteristics and the content of polyphenolic composition in wine. For wine maturation, many types of vessels can be used, with different size and materials, such are stainless steel and wood. Stainless steel is good due to its permeability to oxygen, easier temperature control, and is mainly used for keeping the non-expensive wines prior to bottling. On the other hand, high quality wines usually age in wooden barrels, and during the time spent in wooden barrels wine is exposed to controlled oxygenation and wood compounds are transferred to the wine. Barrel size and temperature influence the wine maturation, a smaller size of barrel, means maturation will be quicker, and if the temperature is lower it will slow the maturation [232]. Macromolecules, which are present in oak wood, belong to the class of polysaccharides (hemicellulose and cellulose) and to the class of polyphenols (lignin), while among other components ellagitannins are the most abundant in oak, there are also some low molecular weight polyphenols and volatile substances. These compounds are extracted into the wine during maturation, and this is the main reason for the choice of this aging technology. During wine maturation in oak barrels, ellagitannins are transferred into the wine, and give aged wine astringency and bitterness sensations [233,234,235], and due to their ability to consume the oxygen they act as antioxidants [236,237]. Ellagitannins also react with anthocyanins forming complexes that are much more stable compared to the grape derived anthocyanins [230,238], they can be also found associated with flavonoids, forming derivatives that have been determined in aged wines [239], and which are also interesting due to their antioxidant activity [240]. The most used, and the most traditional, oak wood belongs to the *Quercus* species from France (*Q. robur* and *Q. petraea*) and from the USA (*Q. alba*) [13]. The concentrations of compounds in oak depend on the type of the oak, i.e., on its drying and toasting conditions, and on the origin of the oak, and there is great variability between the examined species and between the forests [13]. Furthermore, Jeremic et al. [14] tested the antioxidant ability of oenological tannins (procyanidins from grape seed and skin), ellagitannins from oak wood, and gallotannins from gallnut in commercial Chianti red wine, and in a model solution. They concluded that the rate of O_2_ consumption was the highest when ellagitannins were added, representing an effective tool in winemaking when there is a need for instant protection against oxidation, but the effect of ellagitannins on the consumption of O_2_ in wine decreases rapidly with time, which is a limiting factor for their use. While tannins from skin and seed had more consistent reactivity and lower rate of oxygen consumption, gallotannins showed low performance in protection against the oxygen exposure. Besides the addition of tannins into wine in order to improve the concentration of phenolic compounds and antioxidant activity, there are studies that report that the addition of some by-products that appear during winemaking, or derived during making wood barrels, can also be used. The addition of seed from white grape by-products increased the antioxidant activity, phenolic content, and color stabilization of red wine [241,242,243,244]. Jara-Palacios et al. [244], studied the influence of winemaking by-products (seed, stems, skin, and pomace) on the wine antioxidant activity and copigmentation, and concluded that addition of these by-products could improve the wine color and its bioactivity. Escudero-Gilete et al. [245] evaluated the potential use of wood shavings, by-products that appear during wooden barrel production, in order to improve red wine color and antioxidant activity. They used two types of shavings, American and Ukrainian, and concluded that these kinds of cooperage products represent a natural source of copigments and antioxidants, and that Ukrainian shavings provided better color stability.

In beer production, after fermentation, maturation, and lagering, it was found that the content of phenolic compounds was lost by 17%, because of the adsorption to cold trub and yeast [162]. In biological terms beer represents a stable product, but its shelf life is not unlimited, because of its flavor and colloidal stability changes. Vanderhaegen et al. [246] observed that during beer aging, a typical aging aroma will appear, and bitterness sensation decreases. Storage conditions (light and temperature) play an important role in beer aging, and it was noted that these factors influenced the significant decrease of α-acids after 5 months of storage [247]. Besides change in the content of α-acids during beer aging, the content of phenolic compounds also was changed with beer aging. In the research of Li et al. [248] it was determined that the substantial decrease of phenolic compounds occurred within the first three months of aging. Total phenolic content was decreased in examined samples from 16 to 23% within six months of storage, and the antioxidant activity of examined samples behaved in the same way [248]. Even in the 80s of the last century, researchers investigated the changes of polyphenolic compounds during beer production. It was found that after storage of six months, concentrations of the flavanols group ((+)-catechin and (−)-epicatechin), and prodelphinidin and proanthocyanidin B3, decreased [249]. Higher stability of monomeric flavanols was also observed. As was mentioned, during wine aging due to acidic wine conditions and the reaction that occurs among procyanidins, large sized molecules are formed that afterwards precipitate, leading to the decrease of astringency. An important role in these reactions is played by the presence of oxygen [14]. Conditions in beer a medium are less acidic, therefore these kinds of reactions can be performed more slowly, and beer is not exposed to the same levels of oxygen as wine, it is unlikely that procyanindin polymerization will occur during beer storage. Considerable decrease of small flavonoid molecules (monomer to trimers) was observed after the storage of one year at 20 °C [250]. It was also determined that prenylated flavonoids are distinguished by high stability in beer during storage, even after 10 years beer aging at 20 °C, determined concentration of prenylated flavonoids was not significantly different to the concentrations in fresh beer [251]. Heuberger et al. [252] observed that xanthohumol concentration decreased during beer storage, while on the contrary, the concentration of its isomer isoxanthohumol increased. The changes of free trans-piceid and trans-resveratrol, during one year of beer aging at different temperatures, 4 and 10 °C, were investigated by Jerkovic et al. [253], and it was shown that trans-piceid content was not changed, while the content of trans-resveratrol decreased.

Recently, new methods for increasing the content of bioactive compounds in beer, and in the same way of increasing its antioxidant potential have been reported [10,113,254,255]. Particularly, the global rapid growth of the craft beer industry has achieved a huge success, even in the countries that are not recognized as traditional beer producers. Uniqueness and the additional value of the craft beer are due to the addition of innovative raw materials, along with constant main ingredients (malt, hop, water, and yeast). Special hibiscus ale beers, in which different extract concentrations were added, and compared by antioxidant activity, and the content of total phenolic compounds besides other physicochemical changes, during a forced aging process, were analyzed [113]. Analyzing antioxidant capacity and total phenolic content during period of 7 days storage at 45 °C, decrease of both parameters were observed, but it was shown that hibiscus extract is an important source of anthocyanins and phenolic compounds with antioxidant features. Eggplant peel extract was added at the end of the maturation process in order to obtain a high value-added beer, and it was observed that total flavonoid content was stable during whole tested period, while a slight decrease of total phenolic content was noticed after seven days of storage, but even with this slight decrease total phenolic content was significantly higher in beer enriched with eggplant peel extract compared to the control beer. Regarding antioxidant activity, it was found that it rises with addition of eggplant peel extract [10]. These results were in agreement with results obtained by Đorđević et al. [254] who used different extracts of medicinal plants in lager beers, and by Ulloa et al. [255] who investigated the addition of propolis in lager beers. Veljović et al. [256] have shown that it is possible to produce a pleasant, special type of beer using a fermenting mixture of grape must and wort, with higher content of total phenolic compounds in comparison to commercial light beers. In their research, total polyphenolic content in the special types of beer made from grapes, depending on grape variety, different yeast strains, and different wort to grape ratio, was also investigated. They observed the significant impact of grape varieties, content of their addition to wort, and also of the yeast strains, on the total polyphenolic compounds in analyzed samples. For alcoholic fermentation, wine strain *Saccharomyces cerevisiae* and a strain used for brewing *Saccharomyces pastorianus*, were used and higher contents of polyphenols were obtained using wine yeast *S. cerevisiae*. Beer without grape addition, fermented with brewing yeast obtained a mean value of the content of total polyphenols of 95.94 mg/L, while beer with the addition of 30% of Cabernet Sauvignon grapes, which utilized yeast from wine industry, obtained 754.4 mg/L. While among investigated grape varieties (Prokupac, Pinot Noir, and Cabernet Sauvignon), the highest polyphenols content was determined when Cabernet Sauvignon was used, and the lowest in case of Prokupac beer sample; with increasing the amount of added crushed grapes, phenolic content also significantly increased. A recent study by Lasanta et al. [257] investigated the use of five different strains, all of which belonged to *Saccharomyces cerevisiae*, but two for bottom and three for top alcoholic fermentation, varying also the temperatures. Polyphenolic content was higher when lower temperature was applied (12 °C), which was explained by longer fermentation, and at same time longer maceration of these compounds from the used raw material. In addition, the influence of the yeast strain was not shown as significant, particularly at applied lower temperatures. Moreover, recently, the addition of different kinds of fruits (cherry, peach, apricot, raspberry, plum, grape, apple, and orange), and the influence on polyphenolic content and antioxidant activity in beer was analyzed [258]. Most fruit beers obtained a higher antioxidant activity, and total flavonoids and polyphenols content, particularly for beer with the addition of cherries, followed by beers with addition of grape, plum, and orange. All fruit beers have shown enrichment in the content of catechin and quercetin. Polyphenolic content and antioxidant activity were also investigated in beer with addition of mango fruit, and polyphenolic content in mango beer rose, up to even 44% compared to the control beer, which was also in accordance with higher antioxidant activity [259]. These studies indicated improvements in polyphenolic content and antioxidant activity with the addition of different kinds of fruits, and also confirmed better organoleptic features [259].

## 4. Conclusions

Phenolic compounds present in beer and wine have shown high antioxidant and anti-inflammatory features, and in the last decades beneficial effects on human health due to moderate beer and wine consumption have been indicated by many research studies. In this review, a comparative overview of qualitative and quantitative phenolic compound profiles in wine and beer was evaluated. As was expected, due to the different used raw material and technological processes, there are differences between wine and beer in the presence, as well in the concentrations, of phenolic substances. It was shown that some polyphenol classes can only be found in beer (chalcones and flavanones) and other are mainly found in wine (stilbenes, proanthocyanidins), while flavanols and flavan-3-ols are found in similar concentrations in both beverages. Both beverages represent natural fermented products, and minor changes within the growth of raw material and clime conditions, as well as within the used technology, will impact the final chemical composition of these products. Considering the literature data, the obtained results favor wine as the beverage with a higher content of bioactive compounds, particularly phenolic compounds, and as was expected, with higher antioxidant activity. Overall, in order to decide which of these two alcoholic beverages represents the better choice as a functional drink, a lot of parameters should be considered (social occasion, quantity, individual tolerance, etc.). As it was mentioned, drinking pattern is very important, only light-moderate drinking is recommended, and it should not be forgotten that the choice firstly depends on individual preference.

Nowadays, the brewing industry and winemakers put a lot of effort in obtaining a final product that will be unique, with more potent antioxidant activity, and with satisfying sensory characteristics, to attract consumers, who are now more aware of alcoholic beverages influence on human health. Winemakers began even from the vineyard, applying new additives that would improve phenolic composition in the grape, and afterwards, taking care through every step to the final product. After all, from the winemaker’s point of view, the aim is to produce wine that will satisfy all required safety conditions, and with this added value, and at the same time attempting not to increase the costs. A good solution to this, is the use of all by-products, which occur during grape processing and winemaking. In the brewing industry, besides changing hopping regimes and influencing other technology phases, craft breweries that have expanded rapidly all over the world, are doing their best to produce authentic beer, in terms of flavor. Research on using different kinds of fruit in order to obtain special beer with added value, with a focus on sensory improvement and differentiation, has been performed. Both industries should consider changes in clime conditions, and research on new modified technologies is always an open issue, like the use of some by-products and additives within the production.

## Figures and Tables

**Table 1:** Ranges of some phenolic compounds in red wine and beer.

	Red Wine	References	Beer	References
**Cinnamic Acids (mg/L)**				
ferulic acid	0.05–10.43	[30,70,71,72]	0.01–5.04	[12,45,46,73,74,75,76,77,78,79,80,81,82]
*p*-coumaric acid	0.02–8.00	[27,30,70,71,72]	0.003–55.80	[9,12,45,73,78,79]
caffeic acid	0.02–644.50	[27,30,70,71,83]	0.00–23.50	[9,45,46,73,74,75,76,77,78,79,81,82,84]
**Hydroxybenzoic Acids (mg/L)**				
gallic acid	27.10–66.10	[28,71]	0.00–142.20	[9,46,73,74,75,76,77,78,80,81,82]
protocatechuic acid	0.91–1.78	[28,30]	0.01–5.10	[12,75,76,77,78,80,81,82,84]
*p*-hydroxybenzoic acid	2.75–6.20	[28]	0.00–16.84	[12,45,73,75,76,78]
**Stilbenes (mg/L)**				
resveratrol	0,51–11.70	[28,85]	0.002–0.081	[86]
*trans*-resveratrol	0.21–23.00	[27,70,71,87,88,89]	-	-
*cis*-resveratrol	0.01–7.00	[71,87,88]	-	-
total stilbenes	1.00–5.50	[71]	-	-
**Tannins (mg/L)**				
hydrolysable tannins	0.4–50.0	[90,91,92,93,94]	1.5	[81]
**Flavan-3-ols (mg/L)**				
catechin	6.98–91.99	[27,30,36]	0.03–6.54	[12,73,74,75,77,80,81,82]
epicatechin	8.07–52.85	[27,30,36]	0–4.55	[9,74,75,80,81,82]
**Flavones (mg/L)**				
luteolin	0.20–1.00	[95,96,97,98]	0.10–0.19	[82]
apigenin	0.00–4.70	[99]	0.80–0.81	[82]
**Flavonols (mg/L)**				
myricetin	0.70–30.40	[27,30]	0.15–0.16	[82]
quercetin	1.27–65.90	[27,30,36]	0.06–1.79	[74,80,82]
kaempferol	0.61–26.80	[27]	0.10–1.64	[80,100]
**Flavanones (mg/L)**				
naringenin	0.90 to 4.20	[89]	0.06–2.34	[80]

**Table 2:** Ranges of total content of polyphenolic compounds and some phenolic classes, as well as antioxidant activity measured with different analytical tools.

Beer			Wine		
Parameter	Range	Reference	Parameter	Range	Reference
Total polyphenols (FC method); mgGAE/L	127–855	[7,9,12,77]	Total polyphenols (FC method); mgGAE/L	860.2–2912.0	[27,30,31,71]
Total anthocyanogens; mg/L	19.0–84.5	[12,110]	Total anthocyanins; mg/L	21–1011	[71,111,112]
Antioxidant activity; DPPH; mmol TE/L	0.55–6.67	[9,78,113]	Antioxidant activity; ABTS; mmol TE/L	7.5–96.4	[27,30,71]
Antioxidant activity; FRAP; mmol TE/L	0.862–1.271	[12]	Antioxidant activity; FRAP; mmol TE/L	6.9–15.2	[31]
